# Ion-to-electron capacitance of single-walled carbon nanotube layers before and after ion-selective membrane deposition

**DOI:** 10.1007/s00604-021-04805-1

**Published:** 2021-04-02

**Authors:** Elena Zdrachek, Eric Bakker

**Affiliations:** Department of Inorganic and Analytical Chemistry, University of Geneva, Quai Ernest-Ansermet 30, CH-1211 Geneva, Switzerland

**Keywords:** Solid-contact ion-selective electrodes, Ion-to-electron transducer, Single-walled carbon nanotube, Double-layer capacitance

## Abstract

The capacitance of the ion-to-electron transducer layer helps to maintain a high potential stability of solid-contact ion-selective electrodes (SC-ISEs), and its estimation is therefore an essential step of SC-ISE characterization. The established chronopotentiometric protocol used to evaluate the capacitance of the single-walled carbon nanotube transducer layer was revised in order to obtain more reliable and better reproducible values and also to allow capacitance to be measured before membrane deposition for electrode manufacturing quality control purposes. The capacitance values measured with the revised method increased linearly with the number of deposited carbon nanotube–based transducer layers and were also found to correlate linearly before and after ion-selective membrane deposition, with correlation slopes close to 1 for nitrate-selective electrodes, to 0.7 and to 0.5 for potassium- and calcium-selective electrodes.

**Supplementary Information:**

The online version contains supplementary material available at 10.1007/s00604-021-04805-1.

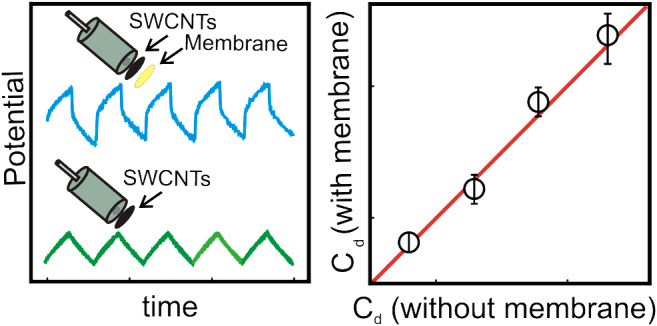

## Introduction

Historically, the need to miniaturize potentiometric sensors gave rise to the development of SC-ISEs and triggered the search for ideal transducer materials. Different transducer materials were described and tested over the years, such as conductive polymers [1–3], nano- and microstructured carbon-based materials [4–9], redox active compounds and mixtures [10–15], as well as several metal-based nanomaterials and composites [16–19].

The potential stability and reproducibility of SC-ISEs are known to be determined by the properties of the ion-to-electron transducer layer. Within this context, the estimation of its capacitance has therefore become an essential part of its characterization, as demonstrated by the fact that 30 of the 46 publications dedicated to SC-ISEs that were cited in a review paper describing progress in the potentiometric sensing in the last 2 years [20] included capacitance estimation results.

The capacitance values can be obtained using various electrochemical techniques such as cyclic voltammetry, electrochemical impedance spectroscopy, or chronopotentiometry. Cyclic voltammetry experiments are less accepted with SC-ISEs owing to the relatively high membrane resistance and because they may trigger undesirable ion-transfer processes resulting in a change of a membrane composition. Therefore, the capacitance of a transducer layer of SC-ISEs is usually determined by means of electrochemical impedance spectroscopy or chronopotentiometry. The latter protocol, introduced by Johan Bobacka in 1999 [2], applies consecutive positive and negative current pulses of the same amplitude to the SC-ISE and monitors the resulting potential drift, and it is often preferred over the former methodology owing to its more straightforward data treatment approach.

Typically, the capacitance of the transducer layer is tested after it is covered with the ion-selective membrane. However, since the transducer materials (i.e., carbon-based [6, 8, 9, 21–23] or metal-based nanomaterials [16–19]) are often deposited manually from a suspension phase, the process is prone to undesirable variability. It would be preferable to test the transducer layer capacitance before the layer is covered with the ion-selective membrane so that the amount of transducer material may be adjusted as needed. Also, avoiding to test the capacitance after the layer is covered with the membrane, which has to be dried and conditioned before testing for transducing layer capacitance, would allow one reducing the time needed to assemble the electrode.

The key condition for this approach to work is that the capacitance value before the layer is covered with the membrane should correlate to that after membrane deposition. In this work, an optimized protocol for the chronopotentiometric estimation of the double-layer capacitance of ion-to-electron single-walled carbon nanotube transducing layers is proposed as a quality control test that can be easily performed at the stage of solid-contact ion-selective electrode (SC-ISE) preparation. Single-walled carbon nanotubes (SWCNTs) functionalized with octadecylamine were chosen for this study, owing to their recurrent use in potentiometric sensors exploited in environmental analysis [21–23] and wearable sensing platforms [24, 25].

## Experimental section

### Reagents

Poly(vinyl chloride) (PVC), bis(2-ethylhexyl) sebacate (DOS), 2-nitrophenyl octyl ether (NPOE), sodium tetrakis[3,5bis(trifluoromethyl)phenyl]borate (NaTFPB), potassium tetrakis[3,5bis(trifluoromethyl)phenyl]borate (KTFPB), tridodecylmethylammonium nitrate (TDMAN), valinomycin (potassium ionophore I), N,N-dicyclohexyl-N′,N′-dioctadecyl-3-oxapentanediamide (calcium ionophore IV), tetradodecylammonium tetrakis(4-chlorophenyl)borate (ETH 500), and tetrahydrofuran (THF) were of Selectophore grade (Sigma-Aldrich, Switzerland). Single-walled carbon nanotubes functionalized with octadecylamine with >80–90% wt. purity (0.5–2-μm length and 2–10-nm outside diameter) were purchased from Sigma-Aldrich (Switzerland). Lithium acetate dihydrate, potassium chloride (>99.5%), sodium nitrate (>99%), and calcium chloride (>97%) were purchased from Sigma-Aldrich (Switzerland). Potassium ferricyanide (>99%) was purchased from ACROS Organics. Aqueous solutions were prepared by dissolving the appropriate salts in Milli-Q water (18.2 MΩ·cm).

### Preparation of ion-selective electrodes

The casting solution for DOS-based potassium-selective membrane was prepared by dissolving 2.3 mg of potassium ionophore I, 0.9 mg of NaTFPB, 5.9 mg inert lipophilic salt ETH 500, 33.5 mg of PVC, and 58.6 mg of DOS in 1 mL of THF; for NPOE-based potassium-selective membranes, 2.3 mg of potassium ionophore I, 0.9 mg of NaTFPB, 33.4 mg of PVC, and 66.1 mg of NPOE in 1 mL of THF; for nitrate-selective membranes, 0.6 mg TDMAN, 1.7 mg inert lipophilic salt ETH 500, 33.7 mg of PVC, and 68.1 mg of plasticizer NPOE in 1 mL of THF; and for calcium-selective membranes, 1.2 mg of calcium ionophore IV, 0.5 mg of KTFPB, 32.9 mg of PVC, and 64.0 mg of plasticizer NPOE in 1 mL of THF.

Commercial glassy carbon electrodes (glassy carbon electrode tip with a diameter of 3.00 ± 0.05 mm, Metrohm (6.1204.300)) were modified with a film of SWCNTs deposited on top of each electrode by drop casting of 20 μL of a SWCNT suspension in THF (1 mg mL^−1^) 1 to 8 times, allowing each layer to dry for 10 min before the next layer deposition. Then, the corresponding membrane cocktail was drop cast on top of the SWCNT film using 3 times an aliquot of 50 μL, and each layer was allowed to dry for 20 min. Finally, the electrodes were conditioned overnight (∼12 h) in a solution of the corresponding salt, namely 10^−4^ M KCl, 10^−4^ M NaNO_3_, or 10^−3^ M CaCl_2_, before measurements.

### Electrochemical equipment and protocols

Potentiometric measurements were carried out with a high impedance input 16-channel EMF monitor (Lawson Laboratories, Inc., Malvern, PA) using a double-junction Ag/AgCl/3 M KCl/1 M LiOAc reference electrode (Metrohm Autolab Utrecht, The Netherlands). Calibration curves for potassium-, calcium-, and nitrate-selective electrodes were obtained by adding successive aliquots of the concentrated solutions of the corresponding salts to Milli-Q water. Activity coefficients were calculated according to the reference [26].

Chronopotentiometric measurements were performed with an Autolab potentiostat/galvanostat PGSTAT 302N (Metrohm Autolab Utrecht, The Netherlands) controlled by Nova 1.10 software running on a PC. A platinum electrode (Metrohm, Switzerland) was used as a counter electrode in the three-electrode cell. A double-junction Ag/AgCl/3 M KCl/1 M LiOAc reference electrode (Metrohm, Switzerland) or an in-house prepared Ag/AgCl wire was used as a reference electrode to perform measurements in aqueous and organic solvent solutions correspondingly. A Faraday cage was employed to protect the system from undesired noise.

The electroactive area of a bare glassy carbon electrode was estimated using the Randles-Sevcik equation:

1 $$ {i}_p=2.69\cdot {10}^5\cdot {n}^{3/2}\cdot A\cdot {D}^{1/2}\cdot c\cdot {\nu}^{1/2} $$

where *D* and *c* are the diffusion coefficient and bulk concentration of the redox probe, respectively, ν is a scan rate, and *A* is an electroactive surface area. For this, cyclic voltammetry experiments at different scan rates (10, 25, 50, 75, and 100 mV/s) were performed with the electrode immersed in an aqueous solution of 0.1 M K_3_[Fe(CN)_6_] and 0.5 M KCl. The slope of the plot of *i*_*p*_ versus *ν*^1/2^ was used to calculate an electroactive area of the electrode according to Eq. 1 assuming the diffusion coefficient value of K_3_[Fe(CN)_6_] to be equal to 7.63 × 10^−6^ cm^2^ s^−1^ [27].

### Analysis of river water sample

The nitrate concentration was determined in untreated Arve River water sample using three types of nitrate-selective electrodes: without any transducer layer and with 2 or 8 layers of SWCNTs as a transducer layer. A standard addition method was used to estimate the concentration of nitrate. In short, the potential was first measured in the river water sample, and then a known volume of standard nitrate solution of 1 M NaNO_3_ was added to the sample and the new potential value was recorded. The nitrate concentration was calculated on the basis of two observed potential values and added concentration of the standard solution according to the following equation:

2 $$ {c}_{sample}=\frac{c_{st}{V}_{st}}{10^{\left(E{}_1-{E}_2\right)/s}\left({V}_{sample}+{V}_{st}\right)-{V}_{sample}} $$

where *c*_*st*_ is a concentration of a standard nitrate solution, *V*_*st*_ is a volume of a standard nitrate solution added to the sample, *V*_*sample*_ is a volume of Arve River water sample, *E*_1_ and *E*_2_ are potential values measured before and after the addition of a standard nitrate solution, and *s* is the potentiometric response slope of the corresponding nitrate-selective electrode.

The concentrations of major anions (Cl^−^, SO_4_^2−^, NO_3_^−^) in the river water sample was confirmed by ion chromatography (Metrohm AG, Switzerland). The river water sample was filtered through 0.2-μm cellulose acetate syringe filter (VWR International GmbH) before injecting into an ion chromatograph. The eluent was a solution composed of 1 mM NaHCO_3_ + 3.2 mM Na_2_CO_3_, along with 50 mM H_2_SO_4_ for regeneration of the suppressor (flow: 0.7 mL min^−1^, pressure: 7.03 MPa, temperature: 45 °C).

## Results and discussion

**Scheme 1:**
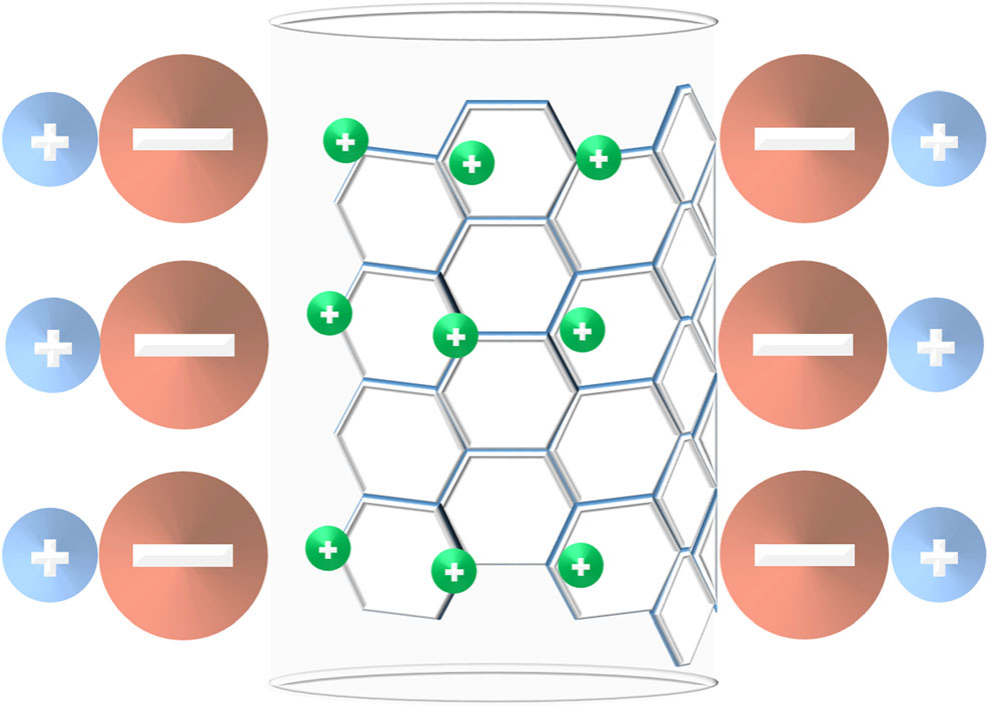
Schematic illustration of an electrical double-layer formation by adsorption of either anions or cations onto the surface of the carbon nanotube

Single-walled and multi-walled carbon nanotubes were introduced as a transducing material for SC-ISEs about a decade ago [6, 28]. It is today commercially available and widely used for potentiometric sensor preparation [21–25]. After deposition on the electrode surface, the carbon nanotubes create a network of well-interconnected fiber-like wires as shown previously by means of environmental [6] and traditional scanning electron microscopy [29]. It is assumed that such a nanostructured material behaves like a capacitor that forms an electrical double layer that stabilizes the electrical potential of the corresponding transducer layer and subsequently the potential of the solid-contact ISE. The electrical double layer is formed between the electrons/holes in the carbon nanotube layer and cations/anions present in a membrane phase as shown in Scheme 1. The latter was originally evidenced by the presence of a large capacitance at the interface of non-functionalized (single- and multi-walled) carbon nanotubes in contact with a background electrolyte solution by means of electrochemical impedance spectroscopy [30]. Further evidence of anion and cation enrichment in the vicinity of the multi-walled carbon nanotube layer was reported by using synchrotron radiation-X-ray photoelectron spectroscopy [31].

In 1999, Johan Bobacka proposed to use chronopotentiometry for capacitance characterization of an ion-to-electron transducer layer underneath an ion-selective membrane [2]. This approach assumes that the SC-ISE can be represented as a *R*_*s*_*C*_*d*_ circuit. When such a circuit is charged by a constant current, the potential is known to increase linearly with time for a current step as follows:

3 $$ E=i\left({R}_s+\frac{t}{C_d}\right) $$

where *E* is the observed potential, *i* is the applied current, *t* is the time, *R*_*s*_ is the resistance element of the circuit, and *C*_*d*_ is a double-layer capacitance.

According to Eq. 3, the observed potential drift is inversely proportional to the capacitance value and thus can be used for capacitance evaluation:

4 $$ Potential\ drift=\frac{\Delta E}{\Delta t}=\frac{i}{C_d} $$

Here, an attempt was made to characterize systematically the transducer material capacitance, directly during its stepwise deposition on the electrode surface. At the same time, our goal was to verify whether there is a correlation between the obtained capacitance values determined without and with an ion-selective membrane. Such a correlation would allow one to estimate and adjust the capacitance of the transducer layer already at the stage of the electrode fabrication that directly translates to an expected capacitance value of the final SC-ISE. To achieve this goal, a double-layer capacitance of the transducer layer before membrane deposition was estimated in acetonitrile solution of 0.1 M tetrabutylammonium hexafluorophosphate (TBAPF_6_) to reasonably mimic an ion-selective membrane phase environment.

A number of modifications of Bobacka’s original protocol are proposed here to improve the robustness and reproducibility of the resulting capacitance values. The first change involves an increased number of measuring cycles to give statistically more relevant results. A single cycle corresponds to the subsequent application of positive and negative current pulses of equal duration. Here, the number of measuring cycles was set to 5, giving 10 capacitance values (2 from each cycle).

**Fig. 1:**
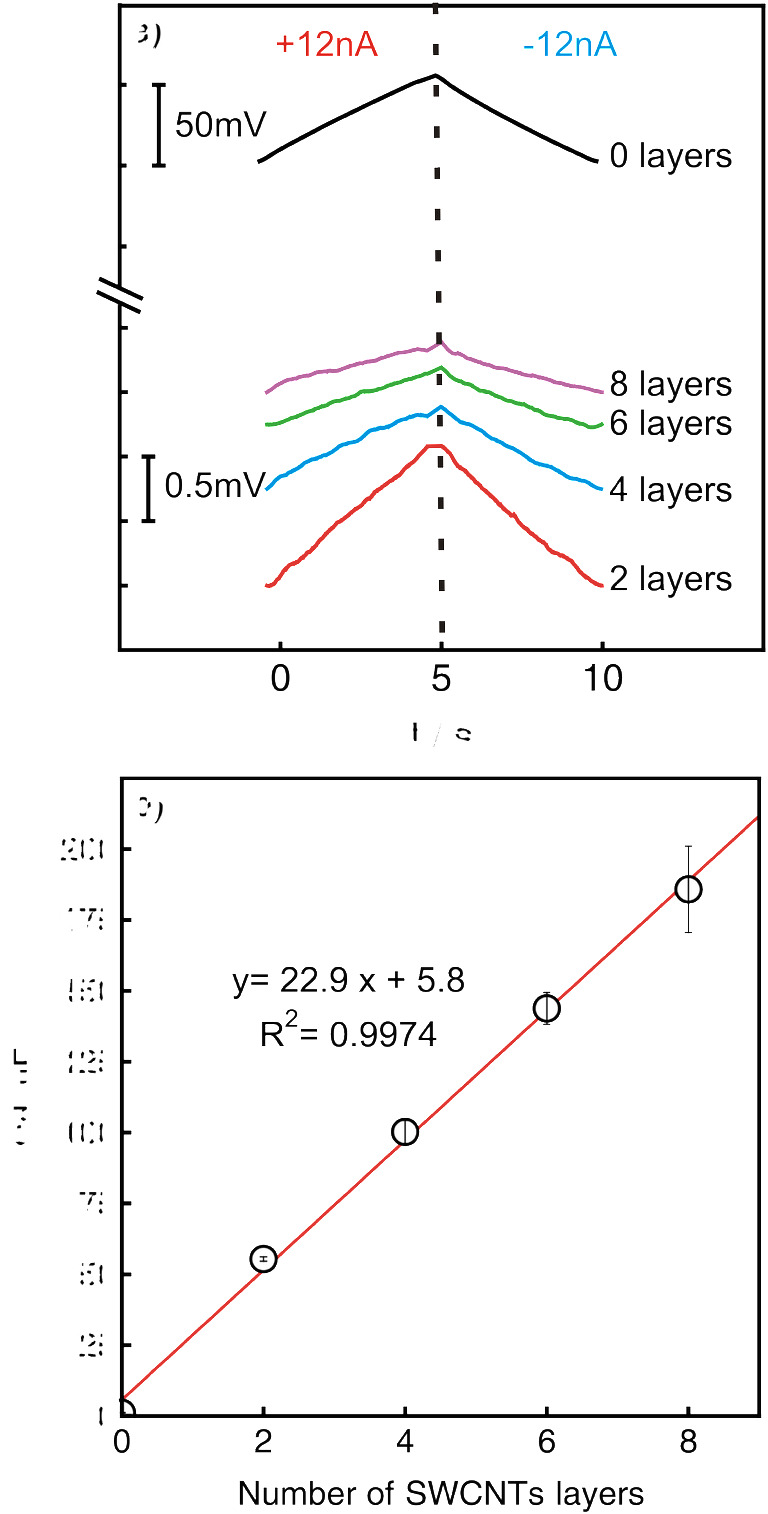
**a** The chronopotentiograms of the 5th measurement cycle observed at ± 12 nA (± 170 nA·cm^−2^) in 0.1 M TBAPF_6_ in acetonitrile for glassy carbon electrode covered with 0–8 layers of SWCNTs. **b** The linear relationship between measured capacitance values and the number of deposited SWCNT layers. Error bars are standard deviations (*n* = 10)

In Fig. 1a, the overlaid chronopotentiograms for the 5th measurement cycle are shown when applying ± 12 nA (± 170 nA·cm^−2^) for 5 s to a glassy carbon electrode (∅ 3.00 mm) with different number of layers of SWCNTs deposited on its surface. One layer of SWCNTs corresponds to 20 μL of 1 mg mL^−1^ suspension prepared in THF. The complete measurements with 5 consecutive cycles are given in Supplementary Information (Figure S1).

Here, the measurements were performed with a single electrode. After each capacitance measurement, the subsequent layer of SWCNTs was deposited on top of an already existing one after the electrode was rinsed with pure acetonitrile and dried for 20 min. From Fig. 1a, the increase of the amount of SWCNTs deposited on the electrode surface results in the expected decrease of the potential drift as a result of the increased double-layer capacitance of the electrode. Consequently, the capacitance value was found to increase linearly with the amount of the deposited transducer material (see Fig. 1b). Papp et al. recently demonstrated a similar relationship [32].

In Figure S1, a shift in potential is shown when moving from one measurement to another, which is becoming smaller with increasing number of SWCNT layers. The potential shift may be explained by a drift of the electrode potential during 60 s of waiting time before starting a new measurement cycle. It can be also concluded that the increase of the capacitance of the transducer layer contributes to stabilization of observed open circuit potential between cycles.

**Fig. 2:**
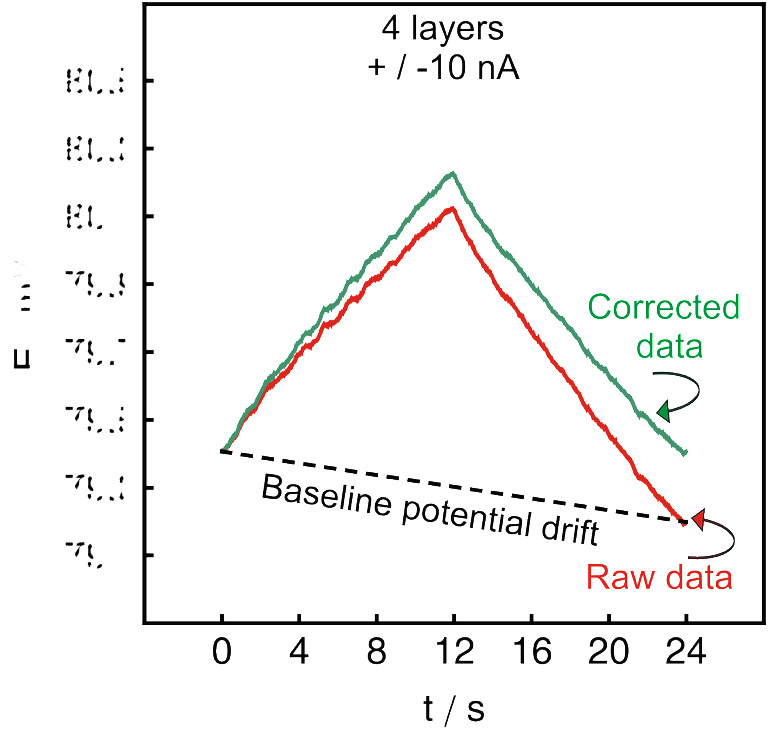
Example of an application of correction for a baseline potential drift. The experimental data represents the chronopotentiogram of an electrode covered with 4 layers of SWCNTs while applying a ± 10 nA (± 141 nA·cm^−2^) current pulse

Clearly, the potential change observed while applying a current pulse may become very small with high capacitance values. To calculate a correct value of the capacitance, it is essential to eliminate or evaluate any additional sources of the potential drift in the system. The latter can be identified by the potential shift of the starting and endpoint of the chronopotentiogram (see Fig. 2).

In our experimental setup, the potential drift of Ag/AgCl element may have caused the observed difference between the initial and final potential values. It is clear that in case of high capacitance values, a strong baseline potential drift may even mask the potential change triggered by the transducer layer. As shown in Fig. 2, the effect of these artifacts was here corrected by subtracting the corresponding value of the baseline potential drift from each obtained data point. In Table S1, one can compare the estimated capacitance values for glassy carbon electrodes covered with 2 layers, 4 layers, and 8 layers of SWCNTs before and after the baseline potential drift correction. One can see that after the baseline correction, the correspondence between capacitance values calculated after applying positive or negative current pulses was improved.

The original protocol applied ± 1-nA current pulses for 60 s, which were chosen to minimize the perturbation of the SC-ISE. However, we noticed that such a small current may result in undesirable random errors and scattering data when the capacitance values of the transducer layer are high. This may be understood by analyzing Eq. 4, as a smaller current results in a smaller potential drift. For relatively high values of the capacitance, it might therefore be difficult to reliably measure the potential change. For this reason, the influence of the current amplitude on the resulting values of the double-layer capacitance was studied. Current values between ± 1 and ± 12 nA (± 14 and ± 170 nA·cm^−2^) were chosen while keeping the total applied charge to ± 60 nC (± 850 nC·cm^−2^) as in the original protocol by appropriately alternating the duration of the applied pulses between 60 and 5 s. This test was performed for three electrodes with different double-layer capacitances: (i) without any transducer layer; (ii) with 4 layers; and (iii) with 8 layers of SWCNTs deposited on its surface.

In Figure S2a, one may see that the capacitance values estimated while applying ± 1-nA (± 14 nA·cm^−2^) current pulses for 60 s give the highest standard deviation values. The uncertainties become larger with increasing double-layer capacitance of the electrode when moving from 0 to 8 layers of SWCNTs. Interestingly, for all three electrodes, the capacitance values were found to decrease with increased applied current amplitude (Figure S2a and its inset). At the same time, the values of the estimated capacitance are reaching a plateau for applied current values between 5 and 12 nA (± 71 and ± 170 nA·cm^−2^) where the difference between the results does not exceed 10%. To verify this plateau region, the influence of the current amplitude on the slope of the relationship between measured capacitance and number of deposited SWCNT layers (0, 4, and 8) was evaluated (see Figure S2b). A similar current amplitude dependence may be easily recognized. The observed slopes change only from 45 to 41 μF per SWCNT layer in the range of 5 to 12 nA (± 71 and ± 170 nA·cm^−2^). To conclude, for a high capacitance material, a ± 1-nA (± 14 nA·cm^−2^) current amplitude cannot be considered optimal. At the same time, current amplitude values between 5 and 12 nA (± 71 and ± 170 nA·cm^−2^) may be recommended for obtaining more reliable and reproducible capacitance values in the range up to 400 μF.

Our proposed protocol includes 5 consecutive measurements cycles with a 60-s pause at open circuit potential before starting a new cycle. Independent of the current pulse duration, this gave an increase of 300 s to the total duration of the measurement. Therefore, to keep the duration of the measurements as short as possible, the same protocol without intermediate pauses was also tested for various current amplitudes. As an example, Figures S3a and S3b show the chronopotentiograms observed at ± 8 nA (± 113 nA·cm^−2^) for glassy carbon electrode covered with 8 layers of SWCNTs with and without a 60-s pause between cycles. One can see the gradual shift in the starting potential for each measurement (about 2.5 mV from the 1st to the 5th cycle) in the case when a 60-s pause was present in the protocol (compare Figure S3a and Figure S3b). This shift can be explained by the potential drift of the electrode at open circuit potential. While the capacitance values obtained by means of both tested protocols were found to be very similar (see Figure S3c), it is apparent that the protocol without pauses allows one to prevent the manifestation of this drift and to shorten the duration of the measurement. Thus, the protocol without pauses was chosen for further measurements.

The final optimized protocol for capacitance estimation was composed of 5 consecutive measurement cycles without intermediate pauses. Three subsequent sets of measurements were performed using 3 different current amplitude values of ± 5, ± 8, and ± 10 nA (± 71, ± 113, and ± 141 nA·cm^−2^), maintaining the same total applied charge for each measurement. Finally, three continuous chronopotentiograms with a time step of 0.01 s were smoothed, corrected for baseline potential drift (see Fig. 2), and analyzed using Wolfram Mathematica software. This protocol allows one to calculate and average 30 values of the double-layer capacitance for each electrode in less than 10 min if the total applied charge value is kept to ± 120 nC (± 1700 nC·cm^−2^).

This optimized measurement protocol was applied for the estimation of the capacitance of the SC-ISEs before and after the deposition of a nitrate- or potassium-selective membrane on top of the SWCNT-based transducer layer. Electrodes with 2, 4, and 8 layers of the deposited SWCNTs were tested and two electrodes of each type were prepared. The measurements before membrane deposition were performed in 0.1 M TBAPF_6_ solution in acetonitrile. Afterwards the electrodes were rinsed with pure acetonitrile, dried for 20 min, and covered with the corresponding ion-selective membrane. After membrane drying and conditioning, the capacitance measurements of ion-selective electrodes were performed in the aqueous solution of ion of interest, e.g., 10 mM NaNO_3_ or 10 mM KCl.

In Figure S4, the results of the capacitance measurements for the same electrodes for a total charge of ± 120 nC or ± 60 nC (± 1700 or ± 850 nC·cm^−2^) before and after nitrate-selective membrane deposition are shown. In all cases, a good linear relationship between the estimated capacitance values and the number of SWCNT layers was observed. The observed capacitance values also correlate adequately with the values from electrochemical impedance spectroscopy for the same transducing material and deposition protocol [33]. It should be highlighted that the value of the total charge ± 120 nC (± 1700 nC·cm^−2^) was preferred over the originally established ± 60 nC (± 850 nC·cm^−2^) owing to the observed better linearity and smaller standard deviation values. The potentiometric response of the prepared nitrate-selective electrodes was confirmed to be close to Nernstian in the concentration range of 3 × 10^−6^–1 × 10^−2^ M which is in good agreement with the literature data for similar membrane composition (see Figure S5) [23]. The latter confirms that an initial exposure of the SWCNT transducer layer to 0.1 M TBAPF_6_ solution in acetonitrile does not negatively affect the potentiometric response of the sensor.

**Fig. 3:**
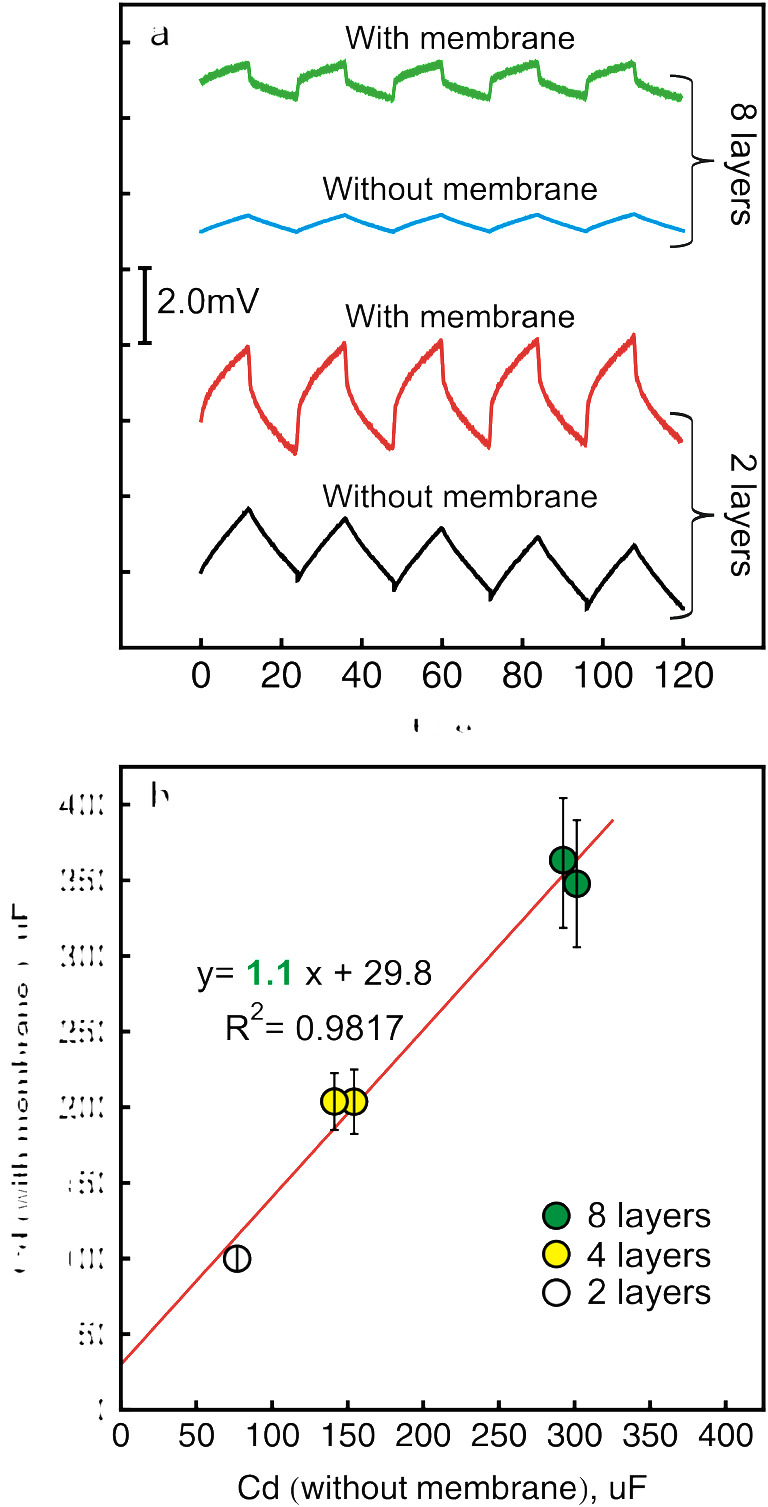
**a** The chronopotentiograms observed while applying ± 10 nA (± 141 nA·cm^−2^) for glassy carbon electrode covered with 2 or 8 layers of SWCNTs with and without nitrate-selective membrane. **b** The correlation between the capacitance values measured before and after ion-selective membrane deposition for nitrate-selective electrode. Error bars are standard deviations (*n* = 30)

The chronopotentiograms observed while applying ± 10-nA (± 141 nA·cm^−2^) pulses to electrodes with 2 or 8 layers of SWCNTs before and after nitrate-selective membrane deposition are presented in Fig. 3a. One may see even with the naked eye that the increase of the potential for this experiment is very similar for the electrode with the same transducer layer before and after depositing the nitrate-selective membrane. The capacitance values of a transducer layer covered with nitrate-selective membrane increase from 100 to 363 uF when moving from 2 to 8 layers of SWCNTs which correspond to the values of 1.9 and 7.0 mF·cm^−2^ if the electroactive surface area of the electrode is taken into account. The calculated electroactive area of three tested bare glassy electrodes was found to be 0.052 ± 0.003 cm^2^.

The correlation between the double-layer capacitance values with and without nitrate-selective membrane is shown in Fig. 3b. The slope of the correlation curve was found to be very close to unity, which was a surprising result. For this case, the measured capacitance value of the transducer before membrane deposition may directly provide a reliable estimate of the capacitance of the final solid-contact nitrate-selective electrode.

To confirm, two potassium-selective membrane compositions with DOS or NPOE as a plasticizer were also studied. The results of capacitance estimation with and without DOS-based and NPOE-based membranes are given in Figures S6 and S8. A linear relationship between the capacitance value and the amount of applied SWCNTs was observed in all cases. The potentiometric response of both types of potassium-selective sensors was confirmed to be close to Nernstian in the concentration range of 1 × 10^−6^–1 × 10^−2^ M (see Figures S7 and S9).

**Fig. 4:**
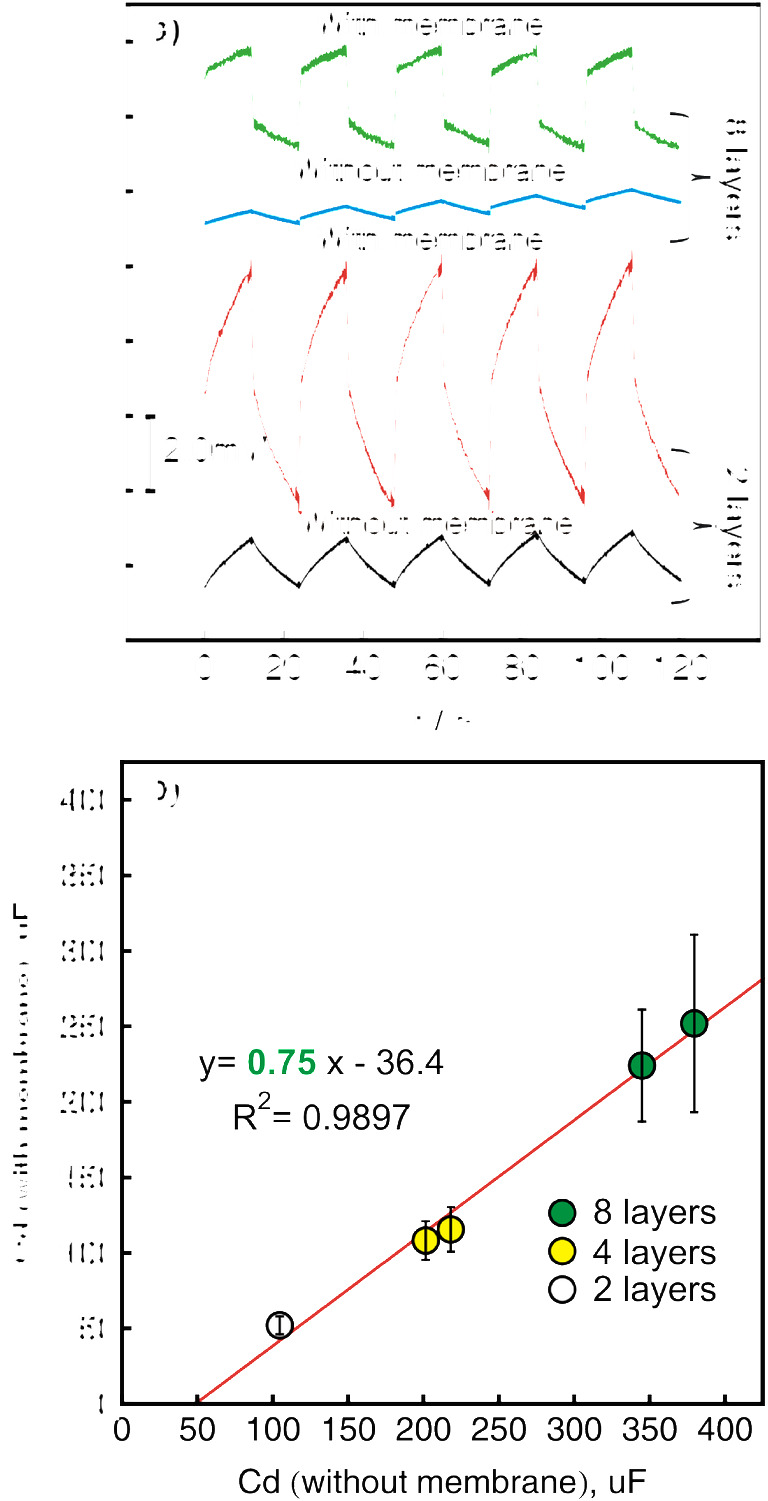
**a** The chronopotentiograms observed while applying ± 10 nA (± 141 nA·cm^−2^) for glassy carbon electrode covered with 2 or 8 layers of SWCNTs with and without potassium-selective DOS-based membrane. **b** The correlation between the capacitance values measured before and after ion-selective membrane deposition for DOS-based potassium-selective electrode. Error bars are standard deviations (*n* = 30)

The capacitance values of a transducer layer covered with DOS-based potassium-selective membrane increase from 52 to 252 uF when moving from 2 to 8 layers of SWCNTs or from 1.0 to 4.8 mF·cm^−2^ accounting for the electroactive surface area of the electrode. In contrast to the NPOE-based nitrate-selective electrode, one may see in Fig. 4a and Figure S10a that the potential increase is steeper on the chronopotentiograms measured after the deposition of potassium-selective membranes based on DOS and NPOE. As a result, the capacitance values observed with potassium-selective membrane are lower than the ones without membrane: the slopes of the correlation curves for the capacitance values observed before and after membrane deposition were below 1, independent of the chosen plasticizer (see Fig. 4b and Figure S10b).

In order to further elucidate the possible reasons of observed difference in the correlation coefficients, the correlation plot was also obtained for calcium-selective electrode prepared with NPOE as a plasticizer. All prepared calcium-selective electrodes demonstrated a close to Nernstian response slope in the concentration range from 10^−6^ to 10^−2^ M independently of the amount of the deposited transducing material (see Figure S11). In a similar manner as for potassium-selective electrodes, it was found that the observed potential change on the chronopotentiograms was bigger after calcium-selective membrane deposition than the ones without membrane (see Figure S12a). The latter resulted in a decrease of the calculated capacitance values after calcium-selective membrane deposition and the slopes of the constructed correlation curves being only about 0.5 (see Figure S12b).

It was suggested earlier in the literature that in a solid-contact ISE, an asymmetric capacitor is established between the SWCNT wall and the solution or the ion-selective membrane [30]. Moreover, it is well-known that a capacitance value of a capacitor constructed of two parallel plates is directly proportional to the dielectric constant of the medium between its plates but inversely proportional to the distance between its plates [34]. Therefore, on one side, one could conclude that a close to 1 correlation slope for nitrate-selective electrode is a result of similar dielectric constant values for NPOE (*ε* = 23.9 [35]) and acetonitrile (*ε* = 37.5 [36]). But in the case of potassium-selective electrodes, the correlation slopes for both electrodes prepared with the polar NPOE or non-polar DOS (*ε* = 3.9 [35]) were equal to 0.7. This strongly suggests that the decrease of the capacitance of the SWCNT layer cannot be explained only by the change in the dielectric constant of the solution/ion-selective membrane matrix. Moreover, taking into account that the correlation slope for NPOE-based nitrate-selective electrode was close to 1 but for NPOE-based calcium-selective electrode it was just 0.5, it may be assumed that the difference in the nature of the ions present in the solution/ion-selective membrane matrix plays a more crucial role.

In case of a solid-contact ISE, the apparent distance between capacitor plates may be determined by the radius of the charge-compensating ions. Considering this, the smaller capacitance of the SWCNT layer covered with potassium- or calcium-selective membrane might be explained by the much larger radius of potassium and calcium ions complexed with ionophores in comparison with TBA^+^ ion present in the solution for estimating the capacitance before membrane deposition.

It should be noted that despite the fact that the slopes of correlation plots are below 1 for potassium- or calcium-selective electrodes, there is an established linear relationship between the capacitance values of the transducer layer before and after the membrane deposition. Therefore, this relationship can be used to predict the value of the assembled solid-contact ion-selective electrodes.

One could see that the capacitance values obtained for the same number of SWCNTs estimated in different experiments are not always the same (compare Figs. 2, 3, and 4). This confirms the difficulty of achieving reproducible transducer layer properties during electrode fabrication when the transducer materials are deposited manually from a suspension phase. It should be noted that the results shown on Figs. 2, 3, and 4 represent experiments performed on different days and with SWCNT suspension freshly prepared in the beginning of each day. The fact that in all three cases the linear relationship between the number of layers and the capacitance values holds suggests that the properties of SWCNT suspension may have been different.

The influence of the transducer layer capacitance on the long-term potential stability of SC-ISEs was also tested for the NPOE-based potassium-selective electrode. As expected, an enhanced potential stability was observed with an increased double-layer capacitance (see Figure S13). The standard deviation of the *E*^0^ value decreases by a factor 3 when one compares SC-ISEs with 2 and 8 layers of SWCNTs while the double-layer capacitance changes from 50 to 250 μF (see Figure S8).

In view of evaluating the performance of nitrate-selective electrodes with varying capacitances of the transducer layer in the presence of common interfering species, a sample from the Arve River was collected and analyzed. Three types of nitrate-selective electrodes were prepared: without any transducer layer and with 2 or 8 layers of SWCNTs as a transducer layer. The results were compared with the values obtained using ion chromatography and are summarized in Table S2. One can see that the potentiometric response of all three prepared electrodes is close to Nernstian. Moreover, it is clear that nitrate concentration determined by all electrodes is very similar. In all cases, the relative standard deviation observed for three measurements did not exceed 2%. A non-significant difference (1–5%) was observed while analyzing the same sample with the reference method of an ion chromatography (see Table S2). Therefore, it may be concluded that the amount of major interfering anions, chloride and sulfate, present in the Arve River water sample at a relatively high concentration according to the results of ion chromatography (345.2 ± 0.9 and 651.4 ± 0.5 μM, respectively) does not influence the quantification of nitrate (see Figure S14a and S14b).

It is not surprising to see that the immediate response characteristics of nitrate-selective electrode without a transducer layer are similar to the ones with 2 or 8 layers of SWCNTs. It is well-known that in the absence of a lipophilic transducer layer, the potential drift and the deterioration of the potentiometric response may manifest itself only with time owing to the formation of the water layer between an electrode surface and the ion-selective membrane [37]. At the same time, a dependence of reproducibility of *E*° value on the capacitance of the transducer layer can be additionally confirmed in this experiment. The standard deviation of observed *E*° value is decreasing while moving from the electrode without any transducer layer to the electrodes with 2 and 8 layers of SWCNTs (see Table S2).

Over the years, many different methods for detection of nitrates in water, such as colorimetry, chromatography, flow injection analysis, electrochemical sensors, biosensors, and optical fiber sensors, were described and reviewed in the literature [38]. All of these methods have different characteristics and sensitivities. At the same time, it is apparent that nitrate-selective electrodes represent a rare class of sensor that allows one to perform a continuous in situ monitoring of nitrate concentration with a detection limit below 1 μM using low-power portable instruments [23, 39].

## Conclusions

The protocol for chronopotentiometric capacitance estimation of SWCNT transducer layer of SC-ISEs was optimized and tested. Current pulses of a higher amplitude (between 5 and 10 nA (± 71 and ± 141 nA·cm^−2^)) and higher total applied charge (± 120 nC instead of ± 60 nC (± 1700 instead of ± 850 nC·cm^−2^)) were found to give more reliable data, especially when the capacitance was relatively high. The protocol with several measurement cycles instead of a single one is also recommended in order to readily assay the reproducibility of the estimated capacitance values.

The optimized protocol was used to estimate the capacitance of the SWCNT layer before and after depositing the ion-selective membrane. Even though the capacitance estimation of the transducer layer was carried out in an organic solvent, a good correlation was found between the obtained values and the ones measured after ion-selective membrane deposition. Interestingly, the slope of the correlation curve was close to 1 for the nitrate-selective electrode while for potassium- and calcium-selective electrodes, it was about 0.7 and 0.5 correspondingly.

This suggests that the capacitance values measured for SWCNT layer before membrane deposition can be used as a direct estimate of the capacitance of an assembled ion-selective electrode. The established correlation allows one to use the optimized protocol as a quality control test at the stage of electrode fabrication when depositing the ion-to-electron transducing layer.

## Supplementary information

The Supplementary Information includes chronopotentiograms with 5 cycles with/without 60-s pause, potentiometric responses, and linear relationships between capacitance values and the amount of SWCNTs for nitrate- and potassium-selective electrodes, results of Arve River water analysis.

ESM 1 (PDF 1.80 mb)

## Data Availability

The data that support the findings of this study are available from the corresponding author upon reasonable request.
