# Supplementary material for: Ion-to-electron capacitance of single-walled carbon nanotube layers before and after ion-selective membrane deposition
Source: Mikrochim Acta. 2021 Apr 2;188(5):149. doi: 10.1007/s00604-021-04805-1 (PMC8018922; doi:10.1007/s00604-021-04805-1)
Supplement: Supplementary file 1 — (PDF 1.80 mb) [file 604_2021_4805_MOESM1_ESM.pdf]

Ion-to-Electron Capacitance of Single-Walled Carbon Nanotube Layers Double Before and After Ion-Selective Membrane Deposition

Elena Zdrachek* and Eric Bakker*

Department of Inorganic and Analytical Chemistry, University of Geneva, Quai Ernest-Ansermet 30, CH-1211 Geneva, Switzerland.

Table of Contents

Figure S1. The chronopotentiograms of 5 measurement cycles observed at +/- 12 nA (+/- 170 nA·cm^{-2}) in 0.1M $TBAPF_6$ in acetonitrile for glassy carbon electrode covered with a) 0 layers, b) 2 layers, c) 4 layers, d) 6 layers or e) 8 layers 4

Table S1. The comparison of calculated capacitance values determined in 0.1M $TBAPF_6$ in acetonitrile for glassy carbon electrode covered with 2 layers, 4 layers and 8 layers of SWCNTs before and after the correction of baseline potential drift 5

Figure S2. a) The influence of the applied current amplitude on the capacitance values estimated in 0.1M $TBAPF_6$ in acetonitrile for glassy carbon electrode covered with 0, 4 and 8 layers of SWCNTs. Error bars are standard deviations (n = 10). b) The slope of the correlation between measured capacitance values and the number of deposited SWCNTs layers (from 0 to 8) plotted as a function of the applied current amplitude. Error bars are standard deviations of the linear regression slope. 6

Figure S3. The chronopotentiograms of 5 measurement cycles observed at +/- 8 nA (+/- 113 nA·cm^{-2}) in 0.1M $TBAPF_6$ in acetonitrile for glassy carbon electrode covered with 8 layers of SWCNTs a) with a 60 s pause and b) without a pause between individual measurements. c) The comparison of the capacitance values estimated by means of the protocol with and without pauses. Error bars are standard deviations (n = 10). 7

Figure S4. The relationship between measured capacitance values and the number of deposited SWCNTs layers obtained for nitrate-selective electrode before and after nitrate-selective membrane deposition while applying the total charge of a) and b) +/- 60 nC (+/-850 nC·cm^{-2}) or c) and d) +/- 120nC (+/-1700 nC·cm^{-2}). Two electrodes were prepared and tested for each type of SWCNTs coating. Error bars are standard deviations (n = 30). 8

Figure S5. The potentiometric response of nitrate-selective electrodes prepared with a) 2 layers, b) 4 layers, c) 8 layers of SWCNTs. Two electrodes were prepared and tested for each type of SWCNTs coating. Error bars are standard deviations (n = 3). 9

Figure S6. a) The relationship between measured capacitance values and the number of deposited SWCNTs layers obtained for DOS-based potassium-selective electrode while applying the total charge of +/- 120 nC (+/-1700 nC·cm^{-2}) a) before and after) potassium-selective membrane deposition. Two electrodes were prepared and tested for each type of SWCNTs coating. Error bars are standard deviations (n = 30). 10

Figure S7. The potentiometric response of DOS-based potassium-selective electrodes prepared with a) 2 layers, b) 4 layers, c) 8 layers of SWCNTs. Two electrodes were prepared and tested for each type of SWCNTs coating. Error bars are standard deviations (n = 3). 11

Figure S8. a) The relationship between measured capacitance values and the number of deposited SWCNTs layers obtained for NPOE-based potassium-selective electrode while applying the total charge of +/- 120 nC (+/-1700 nC·cm^{-2}) a) before and after) potassium-selective membrane deposition. Two electrodes were prepared and tested for each type of SWCNTs coating. Error bars are standard deviations (n = 30). 12

Figure S9. The potentiometric response of NPOE-based potassium-selective electrodes prepared with a) 2 layers, b) 4 layers, c) 8 layers of SWCNTs. Two electrodes were prepared and tested for each type of SWCNTs coating. Error bars are standard deviations (n = 3). 13

Figure S10. a) The chronopotentiograms observed while applying +/- 10 nA (+/-141 nA·cm^{-2}) for glassy carbon electrode covered with 2 or 8 layers of SWCNTs with and without potassium-selective NPOE-based membrane. b) The correlation between the capacitance values measured before and after ion-selective membrane deposition for NPOE-based potassium-selective electrode. Error bars are standard deviations (n = 30). 14

Figure S11. The potentiometric response of calcium-selective electrodes prepared with a) 2 layers, b) 4 layers, c) 8 layers of SWCNTs. Two electrodes were prepared and tested for each type of SWCNTs coating. Error bars are standard deviations (n = 3). 15

Figure S12. a) The chronopotentiograms observed while applying +/- 10 nA (+/-141 nA·cm^{-2}) for glassy carbon electrode covered with 2 or 8 layers of SWCNTs with and without calcium-selective membrane. b) The correlation between the capacitance values measured before and after ion-selective membrane deposition for calcium-selective electrode. Error bars are standard deviations (n = 30). 16

Figure S13. Potentiometric response monitored over 10 days for NPOE-based potassium-selective electrodes prepared with a) 2 layers, b) 4 layers and c) 8 layers of SWCNTs. Error bars are standard deviations (n = 3). 17

Table S2. The results of nitrate detection in Arve river water sample using prepared nitrate-selective electrodes with and without SWCNTs transducer layer compared with the results of ion chromatography (n = 3). 18

Figure S14. a) Overlaid chromatograms for 5 standard calibration solutions containing chloride, nitrate and sulfate. b) A chromatogram of Arve river water sample. The eluent was a solution composed of 1 mM $NaHCO_3$ + 3.2 mM Na_2CO_3, along with 50 mM H_2SO_4 for regeneration of the suppressor (flow: 0.7 mL min^{-1}, pressure: 7.03 MPa, temperature: 45ºC) 19

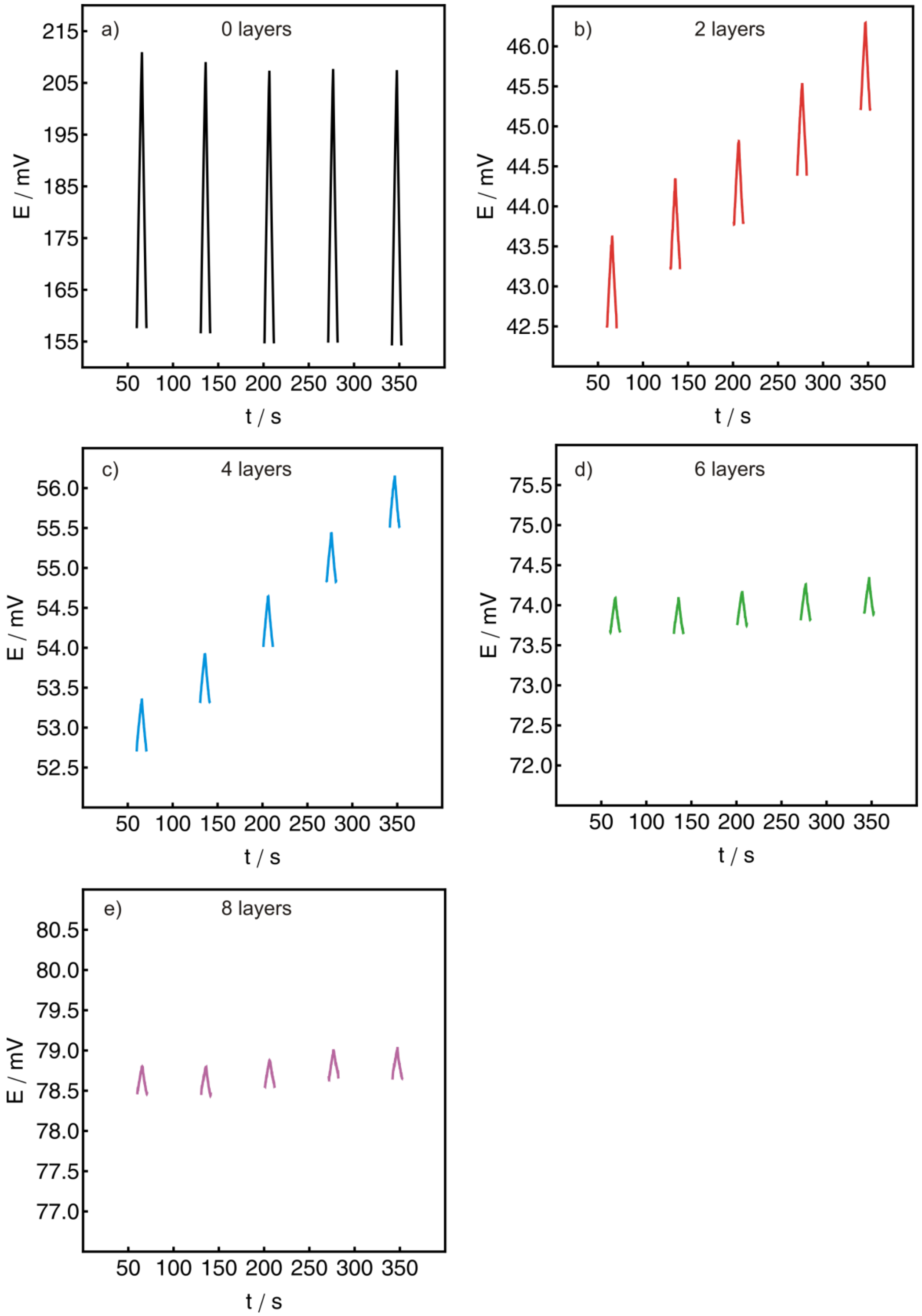

Figure S1. The chronopotentiograms of 5 measurement cycles observed at +/- 12 nA (+/- 170 $nA \cdot cm^{-2}$) in 0.1M $TBAPF_6$ in acetonitrile for glassy carbon electrode covered with a) 0 layers, b) 2 layers, c) 4 layers, d) 6 layers or e) 8 layers

Table S1. The comparison of calculated capacitance values determined in 0.1M $TBAPF_6$ in acetonitrile for glassy carbon electrode covered with 2 layers, 4 layers and 8 layers of SWCNTs before and after the correction of base-line potential drift

Number of layers	Applied current, nA	C_d(before baseline correction)[a], μF	C_d(after baseline correction)[a], μF
2 layers	+10	80.4 ± 0.5	74.2 ± 0.4
	-10	69.0 ± 0.4	74.3 ± 0.5
4 layers	+10	158.9 ± 2.1	135.9 ± 2.3
	-10	117.5 ± 0.9	134.3 ± 1.6
8 layers	+10	288.0 ± 5.6	287.8 ± 2.6
	-10	285.2 ± 4.5	285.3 ± 2.9

[a]The standard deviation values were calculated on the basis of 5 measurement cycles (n = 5)

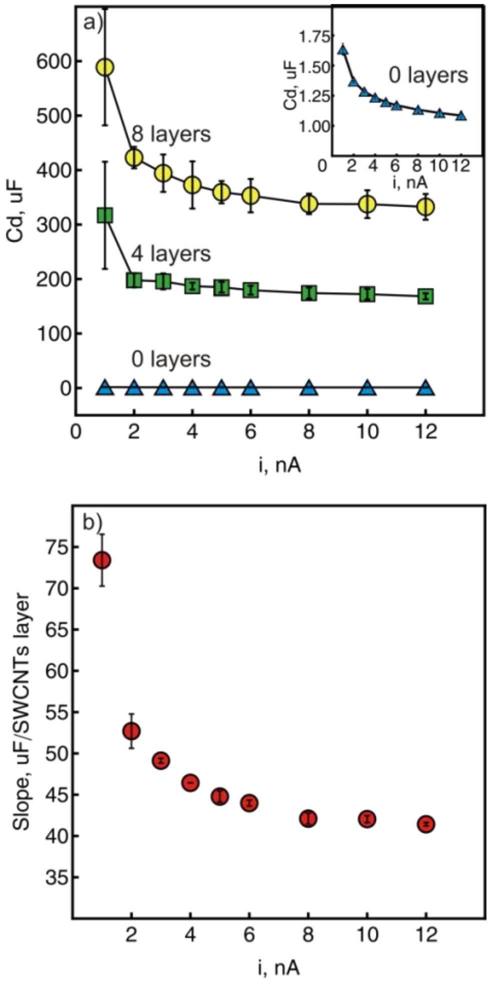

Figure S2. a) The influence of the applied current amplitude on the capacitance values estimated in 0.1M $TBAPF_6$ in acetonitrile for glassy carbon electrode covered with 0, 4 and 8 layers of SWCNTs. Error bars are standard deviations (n = 10). b) The slope of the correlation between measured capacitance values and the number of deposited SWCNTs layers (from 0 to 8) plotted as a function of the applied current amplitude. Error bars are standard deviations of the linear regression slope.

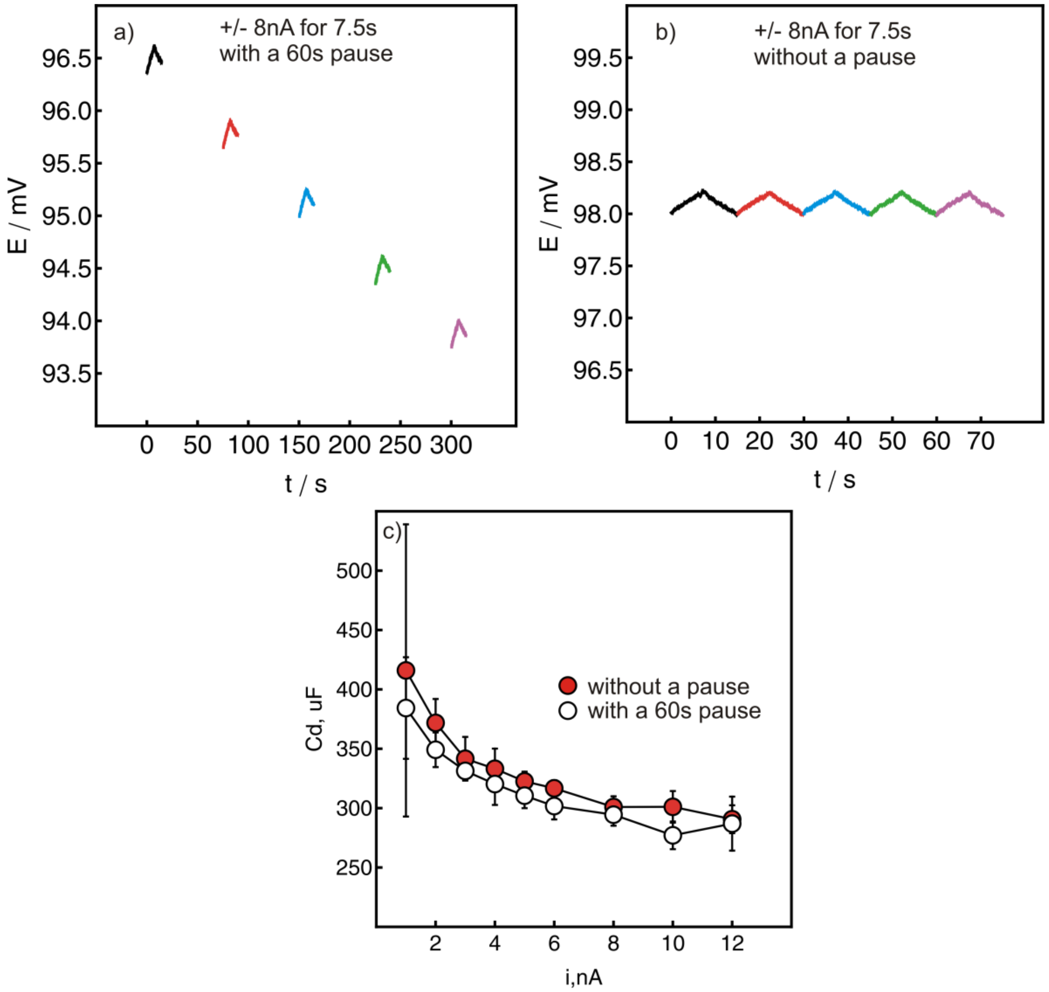

Figure S3. The chronopotentiograms of 5 measurement cycles observed at +/- 8 nA (+/- 113 nA·cm^{-2}) in 0.1M $TBAPF_6$ in acetonitrile for glassy carbon electrode covered with 8 layers of SWCNTs a) with a 60 s pause and b) without a pause between individual measurements. c) The comparison of the capacitance values estimated by means of the protocol with and without pauses. Error bars are standard deviations (n = 10).

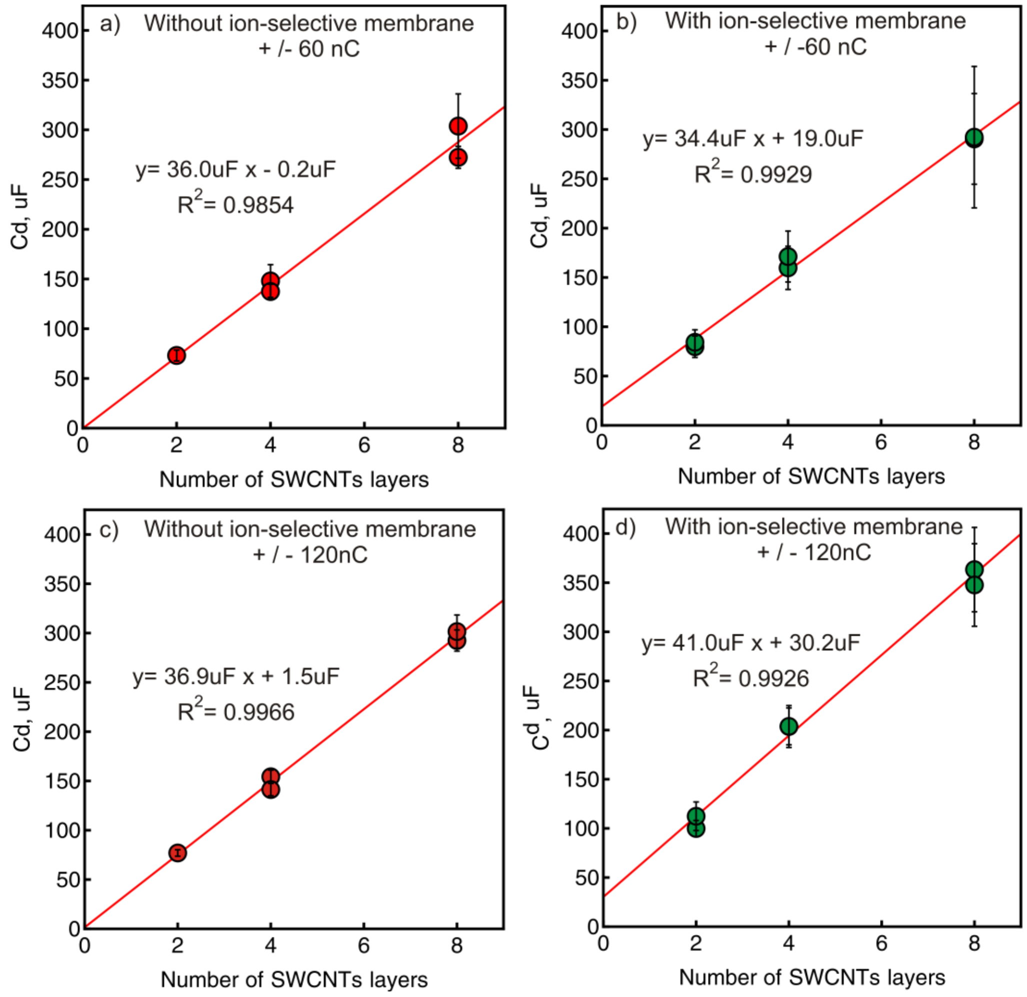

Figure S4. The relationship between measured capacitance values and the number of deposited SWCNTs layers obtained for nitrate-selective electrode before and after nitrate-selective membrane deposition while applying the total charge of a) and b) +/- 60 nC (+/-850 $nC\cdot cm^{-2}$) or c) and d) +/- 120nC (+/-1700 $nC\cdot cm^{-2}$). Two electrodes were prepared and tested for each type of SWCNTs coating. Error bars are standard deviations (n = 30).

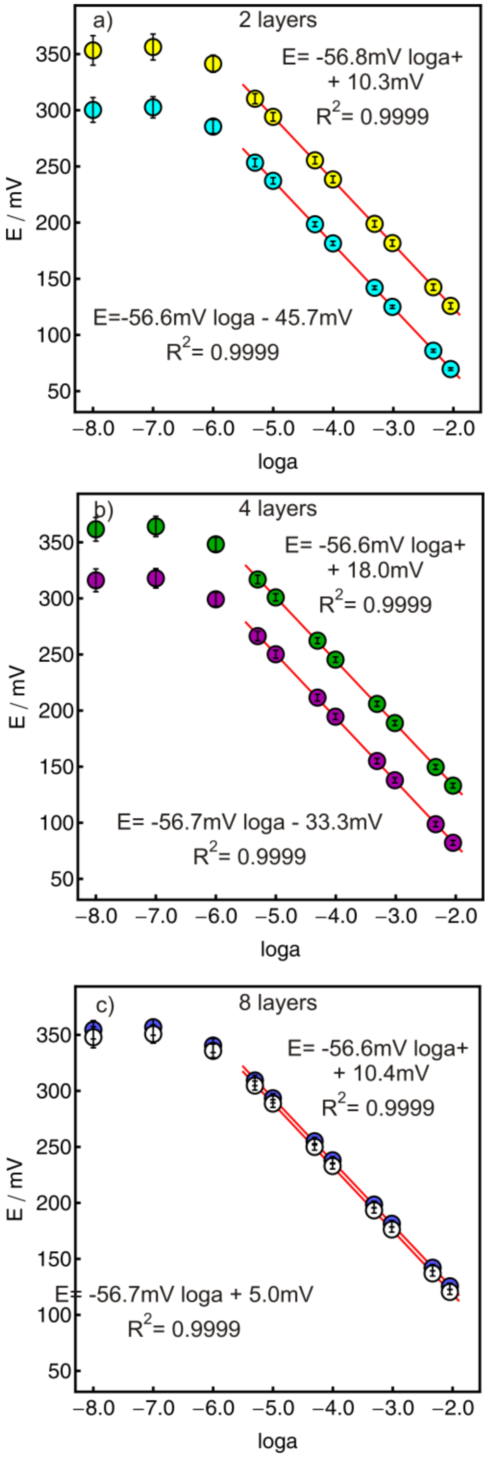

Figure S5. The potentiometric response of nitrate-selective electrodes prepared with a) 2 layers, b) 4 layers, c) 8 layers of SWCNTs. Two electrodes were prepared and tested for each type of SWCNTs coating. Error bars are standard deviations (n = 3).

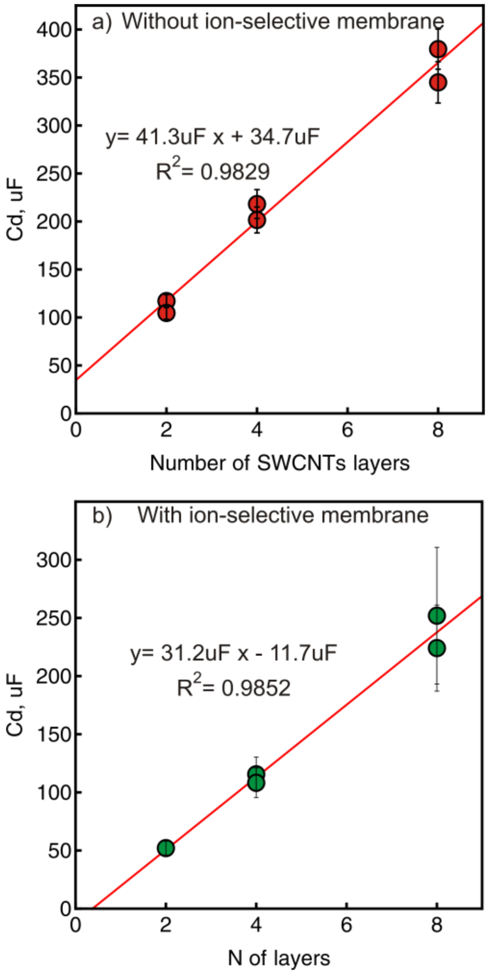

Figure S6. a) The relationship between measured capacitance values and the number of deposited SWCNTs layers obtained for DOS-based potassium-selective electrode while applying the total charge of +/- 120 nC (+/-1700 nC·cm^{-2}) a) before and after) potassium-selective membrane deposition. Two electrodes were prepared and tested for each type of SWCNTs coating. Error bars are standard deviations (n = 30).

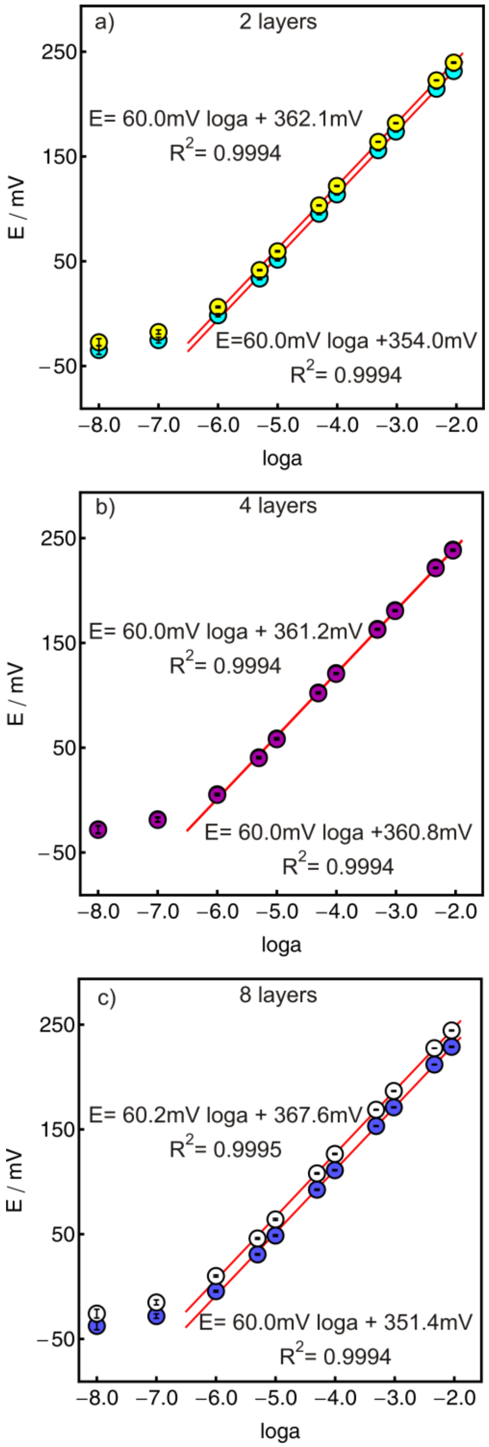

Figure S7. The potentiometric response of DOS-based potassium-selective electrodes prepared with a) 2 layers, b) 4 layers, c) 8 layers of SWCNTs. Two electrodes were prepared and tested for each type of SWCNTs coating. Error bars are standard deviations (n = 3).

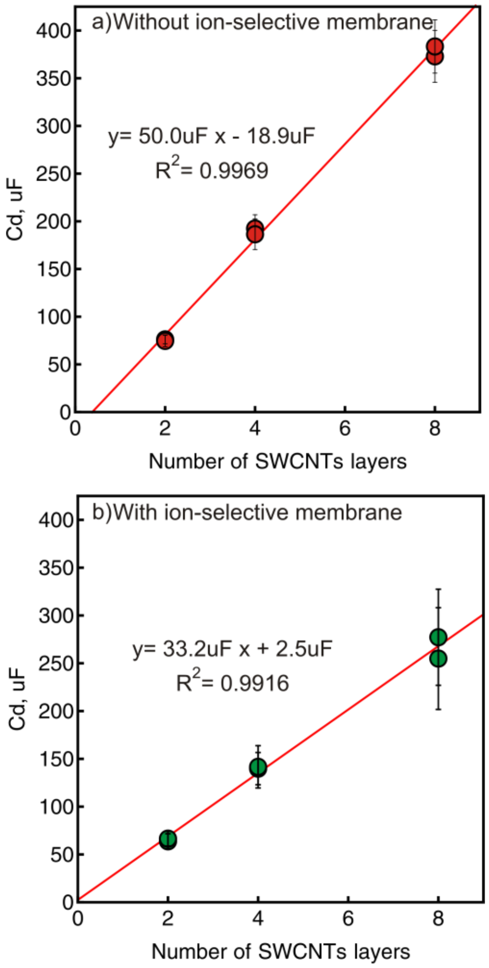

Figure S8. a) The relationship between measured capacitance values and the number of deposited SWCNTs layers obtained for NPOE-based potassium-selective electrode while applying the total charge of +/- 120 nC (+/- 1700 nC·cm^{-2}) a) before and after) potassium-selective membrane deposition. Two electrodes were prepared and tested for each type of SWCNTs coating. Error bars are standard deviations (n = 30).

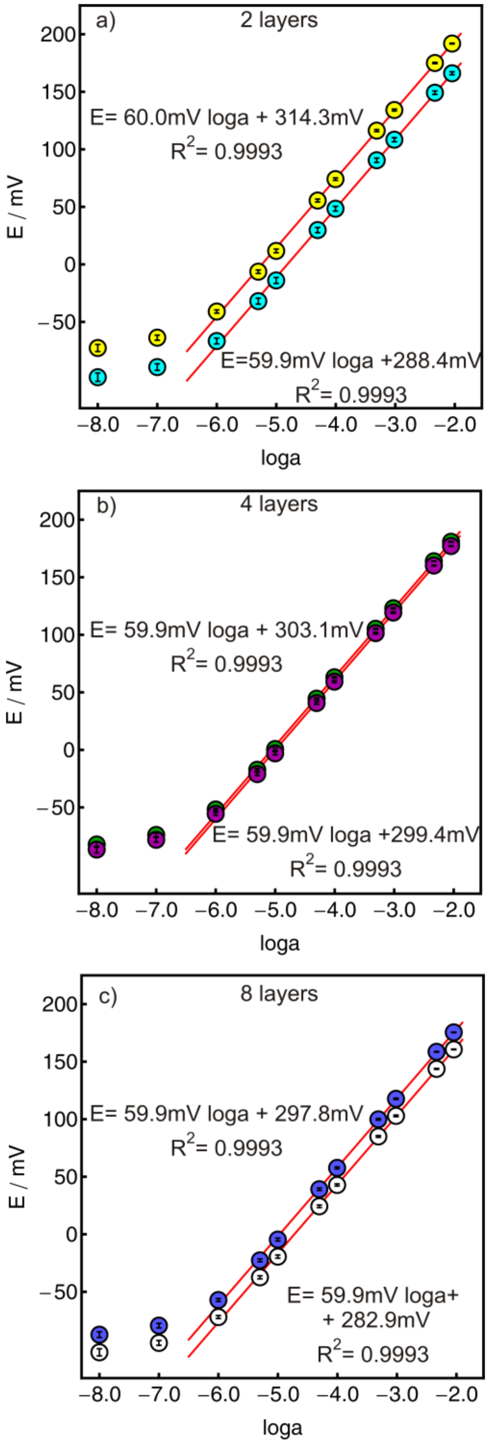

Figure S9. The potentiometric response of NPOE-based potassium-selective electrodes prepared with a) 2 layers, b) 4 layers, c) 8 layers of SWCNTs. Two electrodes were prepared and tested for each type of SWCNTs coating. Error bars are standard deviations (n = 3).

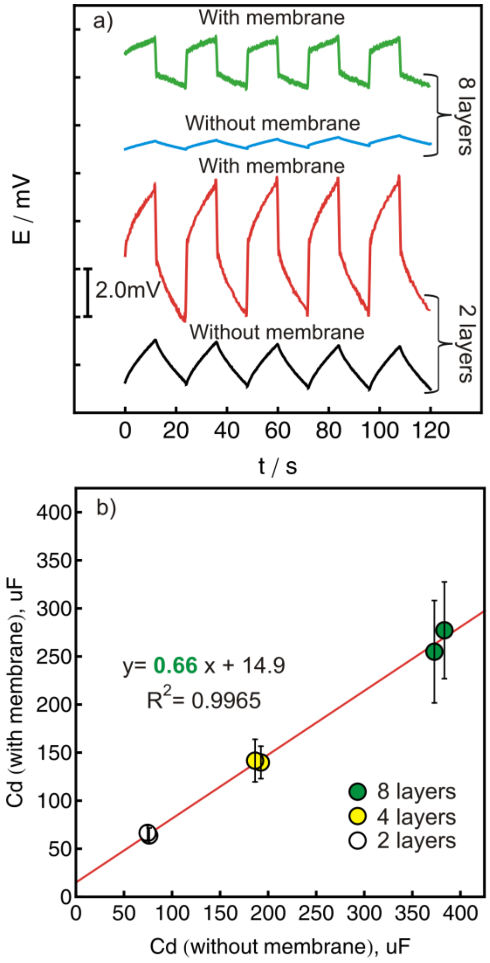

Figure S10. a) The chronopotentiograms observed while applying +/- 10 nA (+/-141 nA·cm^{-2}) for glassy carbon electrode covered with 2 or 8 layers of SWCNTs with and without potassium-selective NPOE-based membrane. b) The correlation between the capacitance values measured before and after ion-selective membrane deposition for NPOE-based potassium-selective electrode. Error bars are standard deviations (n = 30).

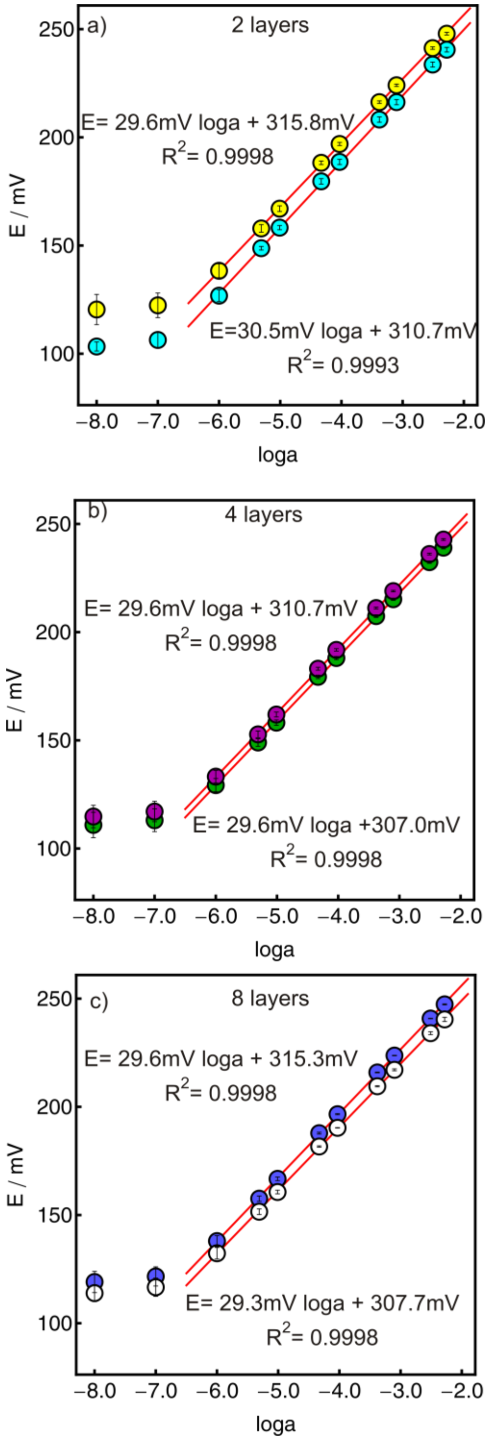

Figure S11. The potentiometric response of calcium-selective electrodes prepared with a) 2 layers, b) 4 layers, c) 8 layers of SWCNTs. Two electrodes were prepared and tested for each type of SWCNTs coating. Error bars are standard deviations (n = 3).

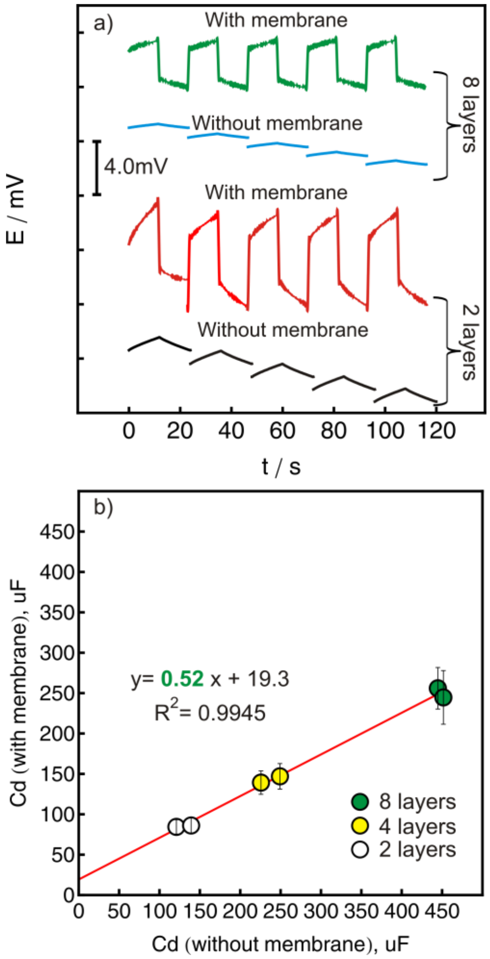

Figure S12. a) The chronopotentiograms observed while applying +/- 10 nA (+/-141 nA·cm^{-2}) for glassy carbon electrode covered with 2 or 8 layers of SWCNTs with and without calcium-selective membrane. b) The correlation between the capacitance values measured before and after ion-selective membrane deposition for calcium-selective electrode. Error bars are standard deviations (n = 30).

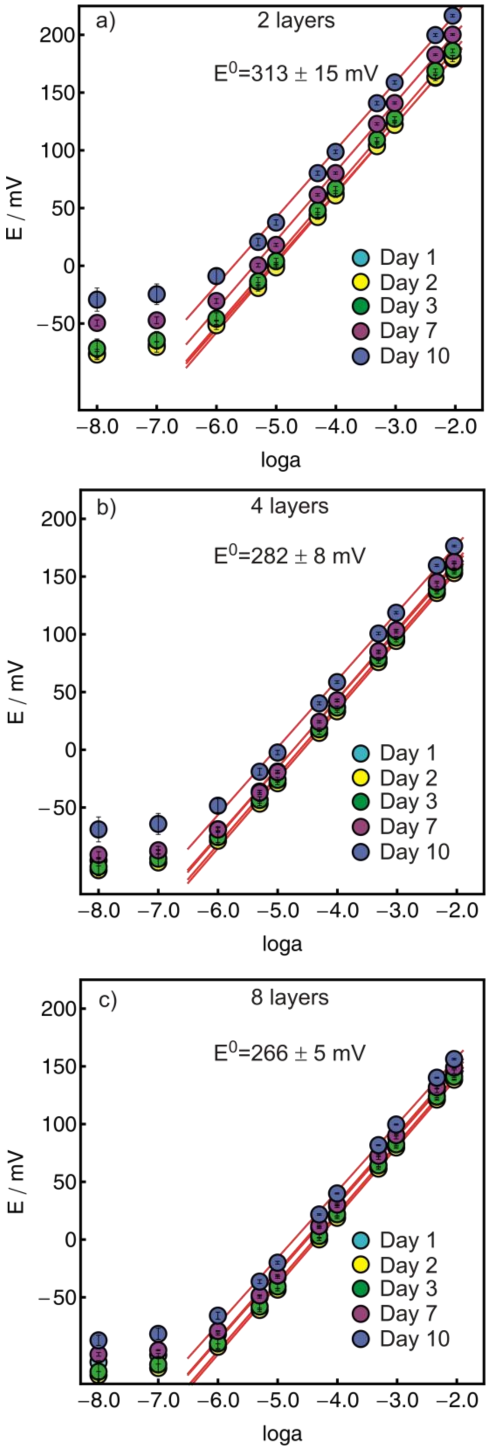

Figure S13. Potentiometric response monitored over 10 days for NPOE-based potassium-selective electrodes prepared with a) 2 layers, b) 4 layers and c) 8 layers of SWCNTs. Error bars are standard deviations (n = 3).

Table S2. The results of nitrate detection in Arve river water sample using prepared nitrate-selective electrodes with and without SWCNTs transducer layer compared with the results of ion chromatography (n = 3).

Number of SWCNTs layers	Potentiometric response function	$c_{NO_3^-}$, µM	
		Potentiometry	Ion chromatography
0	$EMF = (107.1 \pm 9.9)mV - (55.7 \pm 2.9)mV \log a_{NO_3^-}$	75.6 ± 1.2	79.5 ± 0.2
2	$EMF = (104.1 \pm 6.0)mV - (55.9 \pm 0.4)mV \log a_{NO_3^-}$	79.0 ± 2.0	
8	$EMF = (136.3 \pm 0.8)mV - (54.6 \pm 0.3)mV \log a_{NO_3^-}$	76.2 ± 1.1	

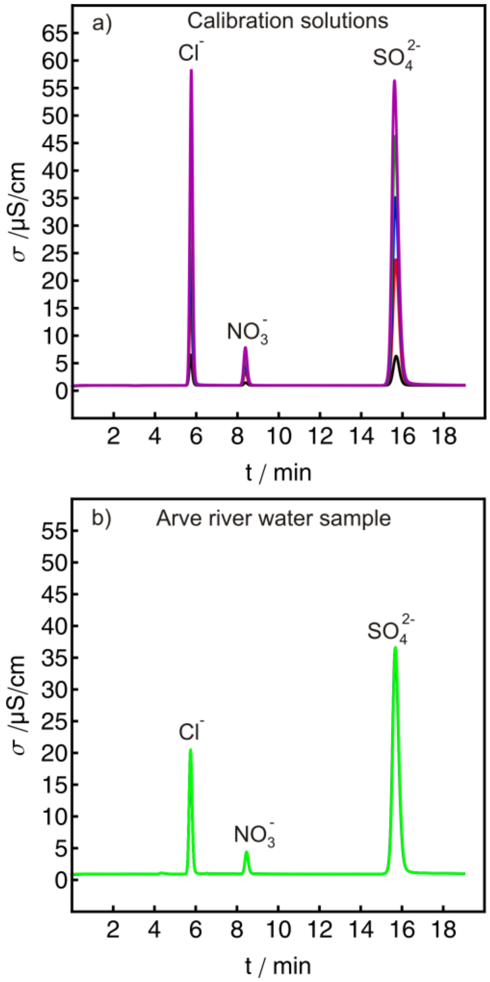

Figure S14. a) Overlaid chromatograms for 5 standard calibration solutions containing chloride, nitrate and sulfate. b) A chromatogram of Arve river water sample. The eluent was a solution composed of 1 mM $NaHCO_3$ + 3.2 mM Na_2CO_3, along with 50 mM H_2SO_4 for regeneration of the suppressor (flow: 0.7 mL min^{-1}, pressure: 7.03 MPa, temperature: 45ºC)
